# Enhancing mental health and well-being in adults from lower-resource settings: A mixed-method evaluation of the impact of problem management plus

**DOI:** 10.1017/gmh.2024.52

**Published:** 2024-04-26

**Authors:** Michela Marchetti, Caterina Ceccarelli, Orso Muneghina, Mara Stockner, Carlo Lai, Giuliana Mazzoni

**Affiliations:** 1 Department of Dynamic and Clinical Psychology and Health Studies, Sapienza University of Rome, Rome, Italy; 2 SOS Children’s Villages Italy, Global Expert Group on Mental Health and Psychosocial Support (GPEG in MHPSS), Milan, Italy; 3 Vrije Universiteit Amsterdam, Amsterdam, The Netherlands

**Keywords:** Problem Management Plus, low and middle income countries, task-sharing intervention, global mental health, WHO

## Abstract

Mental health conditions, recognised as a global crisis, were further exacerbated by the COVID-19 pandemic. Access to mental health services remains limited, particularly in low-income regions. Task-sharing interventions, exemplified by Problem Management Plus (PM+), have emerged as potential solutions to bridge this treatment gap. This study presents an evaluation of the PM+ scale-up in Sub-Saharan Africa (Ethiopia and Benin) and Eastern Europe (Croatia and Bosnia and Herzegovina) as part of a mental health and psychosocial support programming including 87 adult participants. A mixed-method approach assesses the impact of the intervention. Quantitative analyses reveal significant reductions in self-reported problems, depression, anxiety and improved functioning. Qualitative data highlight four main themes: general health, family relationships, psychosocial problems and daily activities. These thematic areas demonstrate consistent improvements across clients, irrespective of the region. The findings underscore the impact of PM+ in addressing a broad spectrum of client issues, demonstrating its potential as a valuable tool for mitigating mental health challenges in diverse settings. This study contributes to the burgeoning body of evidence supporting PM+ and highlights its promise in enhancing mental health outcomes on a global scale, particularly for vulnerable populations.

## Impact statement

This study represents a groundbreaking exploration of Problem Management Plus (PM+) in real-life settings, focussing on Sub-Saharan Africa and Eastern Europe. The choice of these regions is motivated by the unique challenges faced by their populations, including limited access to mental health professionals and a lack of prior research on PM+. This research aims to address critical gaps in the existing literature, specifically its application in non-research settings and the analysis of qualitative aspects. By undertaking a mixed-method evaluation, our study unveils compelling evidence supporting the impact of PM+ in reducing self-reported problems, symptoms of depression and anxiety and improving overall functioning among participants. This efficacy extends across diverse thematic areas such as general health, family relationships, psychosocial problems and daily activities, demonstrating PM+’s versatility in addressing various client needs. The findings underscore PM+’s potential as a scalable approach to mental health challenges in resource-constrained settings. Task-sharing interventions like PM+ emerge as pivotal in bridging the mental health care gap, especially where access to specialised professionals is limited. This study not only addresses the identified gaps in literature but also contributes to the broader discourse on improving mental health outcomes for marginalised and underserved populations globally. In a global context characterised by the exacerbation of mental health conditions, straining resources and disparately impacting vulnerable communities, PM+ provides valuable insights. The study’s implications extend beyond Sub-Saharan Africa and Eastern Europe, advocating for the integration of PM+ into mental health care strategies on a global scale, bringing us one step closer to overcoming the challenges posed by mental health disparities.

## 1. Introduction

Mental health conditions are increasingly recognised as a leading cause of disease burden, affecting millions of individuals worldwide. Recent data from the Global Burden of Disease report revealed that, in 2019 alone, over 970 million people globally were living with a mental health condition (GBD 2019 Mental Disorders Collaborators 2022). The COVID-19 pandemic and associated social restrictions further exacerbated this already dire situation, potentially leading to an additional 76.2 million people developing anxiety disorders (e.g., Santomauro et al., 2021). In line with these findings, growing evidence indicates that social determinants, such as financial strains, food insecurity, forced migration and low social capital, influence both the prevalence and severity of mental conditions (Lund et al., 2018).

Despite the aforementioned evident global mental health needs, the services available to support those requiring support remain insufficient. Financial and human resources for mental health care are overall scarce and unevenly distributed, both across and within countries (World Health Organization, 2021, 2022). This results in a considerable ‘treatment gap’ that disproportionately affects people living in low- and middle-income countries (LMICs) as well as marginalised populations living in high-income countries (HICs) (World Health Organization, 2022). In response to this challenge, innovative ‘task-sharing’ interventions have been developed to increase mental health coverage for mental conditions. Task-sharing interventions involve the delegation of specific mental health tasks or responsibilities from highly specialised professionals to non-specialised individuals, such as community health workers or laypersons, to enhance the accessibility and scalability of mental health care in resource-constrained settings. In task-sharing, trained non-professionals deliver evidence-based psychological treatments under the supervision of specialised mental health workers (Patel et al., 2018). By transferring some mental health care responsibilities from more to less-specialised staff, this approach allows for more efficient support, reaching individuals who might otherwise remain underserved (Hoeft et al., 2018).

One such intervention, developed and promoted by the World Health Organisation (WHO), is Problem Management Plus (PM+). PM+ is a psychological, low-intensity manualised intervention designed for people aged 16 or above who experience symptoms of depression, anxiety or stress, making it ‘trans-diagnostic’ in nature (Dawson et al., 2015), addressing the complexity and comorbidity often observed in mental health conditions, offering a more comprehensive and integrated framework for understanding and treating diverse psychological disorders. The intervention consists of five individual sessions, lasting 90 min, during which clients learn four core strategies (stress management, problem-solving, behavioural activation and skills to strengthen social support) that can help them deal with difficulties faced in their daily lives. PM+ follows the principle of task-sharing; hence, it is delivered by trained non-specialists, called ‘helpers’, who complete an 8-day training, a period of supervised practice, with at least two clients and receive constant supervision by specialised mental health care staff (World Health Organization, 2016). PM+ was initially developed for use in LMICs, in communities affected by adversity. In support of this, several articles (e.g., Dozio et al., 2021) report a significant reduction in post-traumatic symptoms and functional impairment in people living in LMICs. Since its inception, it has been adapted and utilised in different contexts, from communities belonging to LMICs (Sijbrandij et al., 2015, 2016) to communities in HICs (McBride et al., 2021; World Health Organization, 2022; de Graaff et al., 2023). Multiple randomised controlled trials (RCTs) have consistently demonstrated the effectiveness of PM+ in managing practical problems and improving symptoms related to common mental health conditions (CMHCs) (i.e., depression, anxiety, PTSD) among clients at 3-months post-intervention (Bryant et al., 2017; Hamdani et al., 2020; de Graaff et al., 2023).

Evidence from longer-term studies, reporting the effectiveness of the intervention at 12-month follow-ups has been scarce thus far. As evidence of this, Bryant and colleagues (2022), with their robust methodology, in a fully randomised control trial, found that the short-term benefits of this intervention may not be sustained over longer periods.

In addition, qualitative evaluations carried out alongside the effectiveness of RCTs have demonstrated the acceptability and feasibility of this intervention in a wide range of settings (Van’t Hof et al., 2018; Acarturk et al., 2022).

In the wider field of global mental health, the need to understand how interventions work beyond research-controlled settings has become increasingly recognised. Calls have been made to urge researchers to explore the effectiveness of interventions when scaled-up in real-life settings, moving from the research to the implementation space (Jordans and Kohrt, 2020; Murphy et al., 2022). Despite a handful of reports focussing on PM+ during its scale-up, that mostly focus on the early-stage of adaptation, the field still lacks comprehensive data on the effectiveness of PM+ beyond highly controlled research settings (Coleman et al., 2021; Gebrekristos et al., 2021; McBride et al., 2021).

In this work, we aim to contribute to filling this gap through a mixed-method evaluation of PM+ scale-up in Sub-Saharan Africa and Eastern Europe, within the mental health and psychosocial support (MHPSS) programming of the Non-Governmental Organisation (NGO) SOS Children’s Villages (SOS CVI). By collecting evidence on the effect and wider individual-level impact of PM+ in real life, we aim to advance the understanding of whether and how task-shifting interventions can be scaled up beyond research-controlled settings.

## 2. Method

### 2.1. Setting

PM+ was implemented within programmes of the national associations of the SOS CVI federation with individual sessions for all clients. PM+ sessions were delivered in the PM+ supervised practice phase, which followed regional 8-day Training of Helpers (ToHs). Trainers from the global SOS CVI programme on MHPSS conducted the ToHs in Sub-Saharan Africa (Ethiopia and Benin) and Eastern European regions (Croatia and Bosnia and Herzegovina), providing supervision to the trained helpers. Trained helpers delivered the intervention to at least two clients during the phase of supervised practice. Helpers were SOS CVI staff without prior formal training in mental health. Three ToHs sessions took place: one in Bosnia and Herzegovina and Croatia, where 7 helpers received training; one in Ethiopia, where 21 helpers received training and one in Benin, where 26 helpers received training. Then, the intervention was then delivered in the remote mode between May and July in spring 2021 for clients living in Bosnia and Herzegovina and Croatia. On the other hand, in Ethiopia and Benin, the intervention was delivered in person, respectively, in November 2021 and March 2022. The languages in which the ToHs were delivered were English (ToH Bosnia and Croatia; Ethiopia), French and Portuguese (ToH Benin). The trained helpers were provided with PM+ materials in the languages in which the training was provided. The trained helpers then delivered the intervention sessions in their preferred language. Data on the language of delivery of the sessions were not collected.

Helpers participating in the Benin training came from the wider West Africa Region of SOS CVI (i.e., Benin, Cabo Verde, Democratic Republic of the Congo, Ivory Coast, Niger, Liberia, Guinea-Bissau, Cameroun, Burkina Faso, Gambia, Central African Republic, Republic of Guinea). Countries of training delivery are grouped according to the WHO region classification (WHO, 2021). Moreover, this combination was supported by two key indices: economic level and health status. For the economic dimension, we took into account the World Bank income classification (WHO, 2023). Included countries within the European region are categorised as upper-middle income and HIC, whereas those in the Sub-Saharan Africa region fall into the categories of low income (LIC) and lower-middle income countries. Concerning health status, we considered the Healthy Life Expectancy at birth index (WHO, 2023). European countries display an index over 65, while countries in the Sub-Saharan Africa region exhibit an index below 60, indicating important differences in health conditions.

### 2.2. Participants

People aged 16 or above involved in SOS CVI programming and exhibiting some levels of emotional distress were eligible for the PM+ intervention. No specific cut-offs were adopted for inclusion. The term is employed descriptively to denote observable signs or indications of emotional distress, acknowledging the absence of predefined quantitative thresholds for inclusion criteria. This approach allows for a context-specific interpretation of emotional distress without relying on predetermined cut-off values. Participants were excluded when presenting with severe impairment related to a mental, neurological or substance use disorder (e.g., psychosis, alcohol or drug use dependence, severe intellectual disability, dementia) or when at risk for suicide were not eligible for inclusion.

Participants presenting with severe impairment related to a mental, neurological or substance use disorder (e.g., psychosis, alcohol or drug use dependence, severe intellectual disability, dementia) or at risk for suicide were not eligible for inclusion. Participants (87 adults) were residents of the two regions, more specifically 32 from Ethiopia, 12 from Bosnia and Herzegovina, 8 from Croatia and 35 from Benin. The primary spoken languages were Croatian, Bosnian, French, Portuguese, Amharic and English.

All participants completed the 5 intervention sessions; however, only 83 participants carried out the post-assessment measurements (at least two out of the four measurements).

All participants provided informed consent to complete this research.

### 2.3. Measures

As per PM+ manual instructions emotional distress, functioning and self-reported problems were assessed at pre- and post-assessment. Self-reported problems were also assessed during each of the 5 PM+ sessions. Emotional distress was assessed through the Patient Health Questionnaire-9 (PHQ-9) and the Generalised Anxiety Disorder Questionnaire (GAD-7) tools, functioning through the WHO Disability Assessment Schedule (WHODAS 2.0), as indicated in WHO guidelines for the PM+ implementation. Self-reported problems were assessed with the PSYCHLOPS questionnaire. Validated translated versions of the scales were administered to participants based on the country of implementation.

The PHQ-9 is the most frequently used version of the PHQ questionnaire, specifically crafted for assessing the severity of depressive symptoms. It serves dual purposes, applicable for both clinical and research contexts (Kroenke et al., 2001). The latter refers to the English version, for the Portuguese and French versions see, respectively Carballeira et al. (2007) and Lamela et al. (2020).

The tool consists of nine items reflecting distinct symptoms, covering DSM-5 criteria. Respondents are asked to report symptoms referring to a 4-point Likert scale (from “never” to “every day”). In the second section, the functional impairment that depression causes in the normal course of the patient’s life is assessed. Scores range from 0 to 27, a higher score indicates higher depressive symptoms. The PHQ-9 has been adapted and used for use in numerous resource-constrained settings, where it consistently demonstrated good psychometric properties (Carroll et al., 2020).

The GAD-7 is a valid and efficient tool for the screening of general anxiety disorder and the assessment of its severity in clinical practice and research (Spitzer et al., 2006). The latter refers to the English version, for the Portuguese and French versions see, respectively, Souza and colleagues (Sousa et al., 2015) and Micoulaud-Franchi et al. (2016). The GAD-7 is a 7-item scale, where respondents are asked to respond to each statement on a 4-point scale ranging (from “never” to “every day”). Total scores range from 0 to 21, with higher scores indicating higher anxiety symptoms. Similarly, to the PHQ-9, the GAD-7 has been widely adopted in settings of various income levels (Plummer et al., 2016).

The 12-item WHODAS 2.0 is a tool developed by the WHO aimed at generically assessing the health and disability of clients (Ustün et al., 2010). The latter refers to the English version, for the Portuguese and French versions see, respectively, Moreira et al. (2015) and Hoehne et al. (2017). Scale totals can be calculated through a simple scoring method, where the final score ranges from 12 to 60. A higher score indicates a higher loss of function. The 12-item WHODAS 2.0 has been adopted across contexts, demonstrating good reliability and internal consistency (Saltychev et al., 2021).

The PSYCHLOPS is a self-reported tool aimed at capturing the practical problems for which a client is seeking help (Ashworth et al., 2004). The latter refers to the English version. For the Portuguese and French versions, see http://www.psychlops.org.uk/versions. It comprises four items that capture (a) problems, (b) functioning and (c) well-being through Likert and free-text response options. When administered at post-assessment, it includes an additional overall evaluation of well-being. PSYCHLOPS scores are obtained by summing up the Likert items (range 0–20). The scale has been adapted and demonstrated good psychometric properties across populations and countries (Sales et al., 2023). The qualitative data on PSYCHLOPS, in Portuguese and French, were translated into English independently by the first and third authors, during data analysis.

### 2.4. Data analyses

#### 2.4.1. Quantitative data analyses

All the quantitative analyses were performed using RStudio (“RStudio Team”, 2023). All proposed measures were systematically compiled by the helper in the presence of the client during the sessions. The analysis was carried out at the completion of the treatment, thus following the completion of the various measures in the post-assessment. Participants who did not complete the fifth PM+ session were excluded from the research. Since the percentage was very low, missing data were handled according to the regression model in RStudio (*lm* function from *stats* package).

T-tests and regression models with post hoc analyses (Bonferroni) were used to assess the changes in scores in PSYCHLOPS across pre-assessment, sessions 1–5 and post-assessment, were run. The effects of time of measurement (time points) and region of origin were assessed. Four *t* tests, one for each measure (PHQ-9, GAD-7, WHODAS, PSYCHLOPS) were performed. The results of the descriptive analysis of the average test scores for the psychological dimensions investigated will also be presented.

#### 2.4.2. Qualitative data analyses

Free-text responses from the PSYCHLOPS measure, describing the problems experienced by clients and their impact on their functioning, were analysed thematically. Following the protocol, in the pre-assessment phase, each participant produced three open-ended answers to the following questions:

1. Choose the problem that troubles you most.
2. Choose another problem that troubles you.
3. Choose one thing that is hard to do because of your problem (or problems).

English was chosen as the language for analysis; all data collected in other languages (i.e., Portuguese and French) were translated. The Braun and Clarke (2006) approach was used to identify, analyse and report themes to provide a detailed and complex evaluation of the collected data. First, familiarisation with the data was performed. Then, the initial codes of the entire data set were generated in a systematic fashion. Codes (e.g., mental health; physical health) that addressed the research aims under investigation were chosen. The qualitative data analysis tool Quirkos was used to help organise codes and to merge and connect them (Quirkos, 2017. The codes were generated through a collaborative effort involving the first and third authors. The tool is a qualitative data analysis software designed to assist researchers in organising, analysing and interpreting qualitative data such as text, audio and video. We imported our qualitative data and created nodes (codes or themes). This process helps in systematically categorising and organising the data. While the overall process was collaborative, individual researchers were assigned specific tasks to maintain independence and rigour in code development. Each researcher independently reviewed the data, identified patterns and proposed initial codes. Through this iterative process, a consensus was reached on the final set of codes. This approach ensured a comprehensive and nuanced understanding of the data. The determination of themes and sub-themes followed a systematic approach. Initial codes were grouped based on shared characteristics, leading to the emergence of overarching themes. Subsequently, these themes were refined through discussions and consensus-building sessions among the research team. To enhance the credibility and reliability of the coding process, an inter-coder reliability check was conducted. This involved cross-checking and discussing coded data points among researchers to validate the consistency of the interpretation and application of codes.

For the textual analysis, we used an inductive approach, thus starting from the data, we created the themes without relying on preconceived categories.

#### 2.4.3. Mixed-method analyses

Regression analyses were run to test whether the impact of the intervention depended on the main thematic areas identified by clients and if differences in reported themes were observed across regions.

## 3. Results

### 3.1. Participants

Then, 87 adults (18 males) with a mean age of 36 years (SD = 11.6) (67 from Sub-Saharan Africa and 20 from Eastern Europe) received the complete PM+ intervention. Twenty-six participants (31%) reported being single, twenty-six (31%) married and fourteen (16%) divorced. Most participants were reportedly employed in a paid job (N = 35, 41%) or were self-employed (N = 13, 15%); the remaining participants were in a precarious or absent employment situation. Finally, 14 out of 87 (16%) participants stated that they had received previous mental health treatment. See Table 1 for a complete summary of the participant demographics.

**Table 1.** Summary of included clients’ demographics

		Country		Total
		Sub-Saharan Africa	Eastern Europe	
Gender	Male	15	3	18 (19%)
	Female	52	17	69 (81%)
Mean age	Male	33 years	42 years	/
	Female	37 years	37 years	/
Relationship status	Single, never married	23	3	26 (31%)
	Married	16	10	26 (31%)
	Separated	4	0	4 (5%)
	Divorced	10	4	14 (16%)
	Widowed	6	1	7 (8%)
	Long–term romantic relationship/cohabitating	4	2	6 (7%)
	Not known	2	0	2 (2%)
Job status	Paid work	26	9	35 (41%)
	Self–employed	12	1	13 (15%)
	Non–paid work	1	0	1 (1%)
	Keeping house/homemaker	4	3	7 (8%)
	Retired	0	1	1 (1%)
	Student	8	2	10 (12%)
	Unemployed (health reasons)	4	1	5 (6%)
	Unemployed (other reasons)	6	2	8 (9%)
	Other (specify)	4	1	5 (6%)

### 3.2. Quantitative data

The test scores (mean and standard deviation) for the psychological dimensions investigated show a decrease from pre-assessment.

A first regression model with the scores obtained from the PSYCHLOPS evaluation as the dependent variable and time of measurement and region as the independent variables was run. The results showed that both time of measurement (β = −11.87, SE = 0.64, t = −18.58, *p* < 0.001) and region (β = −4.39, SE = 0.89, t = −4.96, *p* < 0.001) were significant predictors of PSYCHLOPS scores and the interaction between time of measurement and region was statistically significant (β = 3.06, SE = 1.25, t = 2.46, *p* < 0.05).

**Figure 1.**
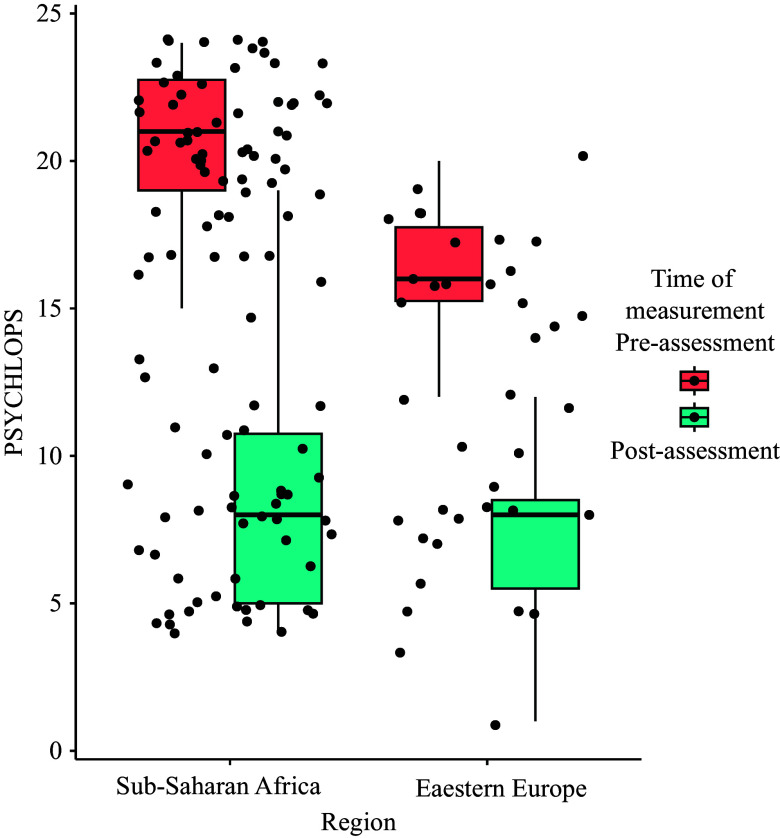
PSYCHLOPS scores at pre- and post-measurement in both regions.

For both regions, Sub-Saharan Africa (t(48) = 18.13, p < 0.001) and Eastern Europe (t(17) = 11.538, p < 0.001) there was a significant decrease between pre- and post-assessment (see Figure 1). However, further *t* tests showed a significant difference between regions at pre-assessment (t(32.49) = 7.51, p < 0.001), indicating that problems reported by clients from European regions were less severe compared to those from Sub-Saharan regions. Nevertheless, such difference was found to not persist at the post-assessment (t(46.99) = 1.44, p = 0.16).

**Figure 2.**
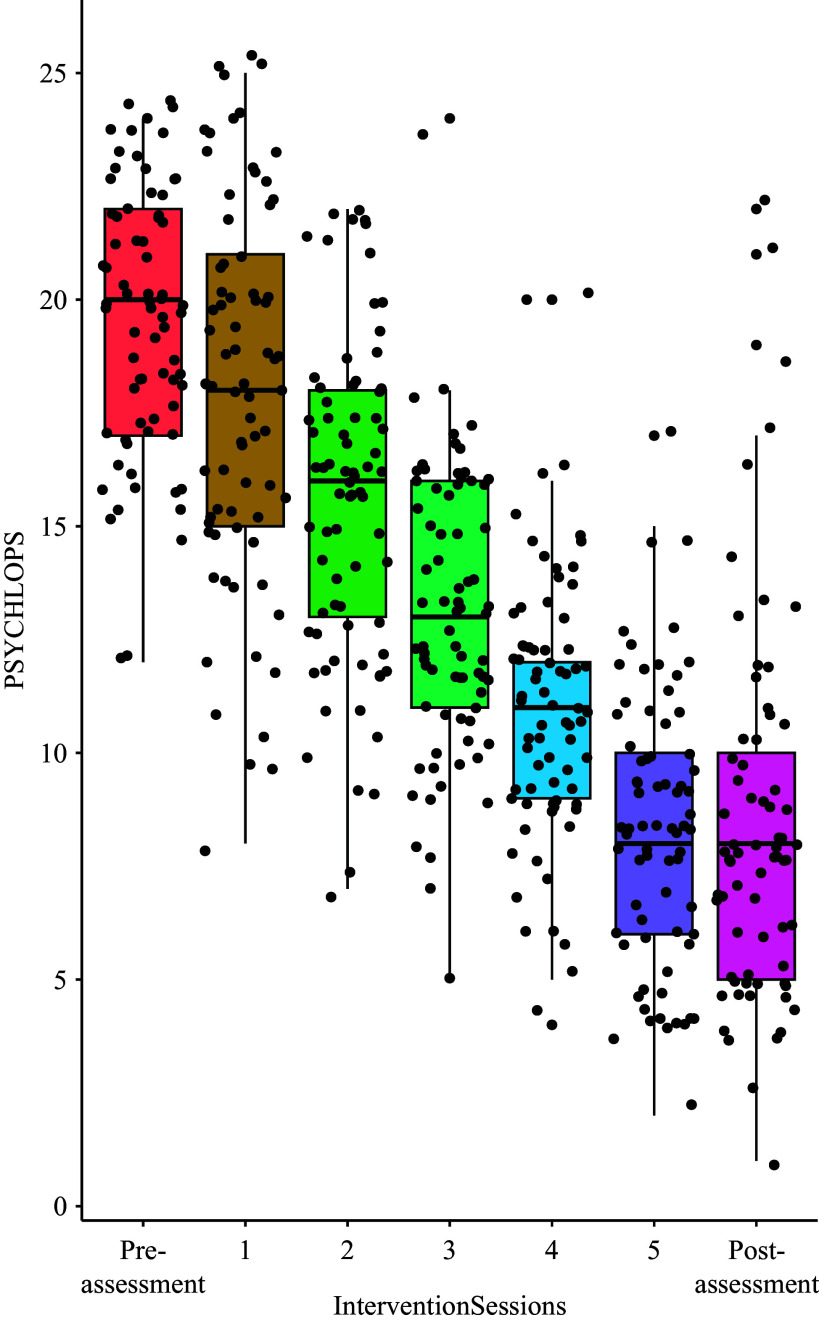
Post hoc PSYCHLOPS sessions.

The results of Bonferroni post hoc analysis (Figure 2) indicate that there was no significant difference between the pre-assessment and the first session (β = 1.53, *p* = 0.15) and between the fifth session and the post-assessment (β = −0.09, *p* = 1.00). However, among all PM+ sessions, significant decreases in PSYCHLOPS scores were observed (Sessions 2–3: β = 2.50, *p* < 0.001; Sessions 3–4: β = 2.50, *p* < 0.001; Sessions 4–5: β = 2.01, *p* < 0.01; Sessions 5–6: β = 2.68, *p* < 0.001).

*T*-tests to compare the difference in self-reported symptoms of depression, anxiety and functioning at pre versus post-assessment showed that the mean scores (i.e., reported symptoms) consistently and significantly decreased between pre- and post-assessment for all reported measures (see Table 2 for means and confidence intervals) (PHQ-9 t(67) = 11.67, *p* < 0.001 [95% CI = 7.9, 11.3]; GAD-7 t(63) = 11.27, *p* < 0.001 [95% CI = 6.0, 8.6]; WHODAS t(63) = 9.65, *p* < 0.001 [95% CI = 10.7, 16.2]).

**Table 2.** Overall means (SD) and confidence intervals; t-tests pre- and post-assessment

Measure	Time of measurement								*t*-tests
	Pre-assessment					Post-assessment			
	*N*	*M*	*(SD)*	(95%CI)	*N*	*M*	*(SD)*	(95%CI)	t(df), *p*
PHQ–9	76	18.63	8.21	18.1, 21.5	71	8.34	4.11	8.9, 11.3	11.67(67), *p* < 0,001
GAD–7	72	15.72	5.68	14.2, 16.9	65	6.54	2.50	7.1, 8.7	11.269(63), *p* < 0,001
WHODAS	73	34.42	11.61	29.1, 34.4	70	17.63	4.11	16.3, 19.6	9.6478(63), *p* < 0,001
PSYCHLOPS	73	19.30	3.34	18.9, 20.3	70	7.76	3.86	7.5, 9.4	20.303(66), *p <* 0,001

### 3.3. Qualitative data

Analysis of the free-text responses from the PSYCHLOPS measure revealed four key main themes.

More specifically, client’s concerns related to general health, family relationships, daily activities and psychosocial problems (see Table 3 for specific sub-themes).

**Table 3.** Themes and sub-themes

Themes	Sub-themes
General health	Mental health Physical health Abuse
Family relationships	Parenthood Partner Family problems Death
Psychosocial problems	Financial problems Community problems Pregnancy problems Legal problems
Daily activities	Work School

#### 3.3.1. Theme 1: General health

Participants reported concerns about their health a total of 147 times. Specifically, 99 PSYCHLOPS free-text responses related to mental health, 40 to physical health and 8 to an episode of abuse. For each quote will be given a unique code in brackets, consisting of gender (M or F), region (A or E) and number representing the order of appearance in the dataset (i.e., ME14).

Regarding mental health, the issues that clients most emphasised were stress, anxiety and lack of motivation. For example, participants often reported phrases such as *“stressed and anxious all the time with the problems I have”* (FA18), *“no motivation to do anything at home and outside the house”* (MA1). On the other hand, the most common problem describing the clinical health condition concerned sleeping difficulties (sometimes alongside insomnia), and other clinical conditions such as heart problems, disabilities or substance abuse (*“I can’t sleep. I have insomnia and nightmares.”* [FA18]; *“take substances, feel hopelessness, problem of keeping hygiene”* [MA86]). Finally, with regard to cases of abuse, two clients reported becoming pregnant as a result of the violence they suffered (e.g., *“Raped and made pregnant, her boyfriend abandoned her”* [FA35]).

#### 3.3.2. Theme 2: Family relationships

Participants reported a total of 121 times concerns about their family relationship. Specifically, 40 sentences related to the relationship with (ex)partners, 35 to parenthood, 38 to family problems and 8 to the death of a family member. Family relationships, especially with partners, appeared to be one of the main problems reported by clients. Communication problems within the couple were commonly mentioned (e.g., *“my marriage is very complicated”* [FA3]*, “he and his wife do not talk to each other”* [MA23]). Another re-occurring theme was that of divorce, with subsequent changes in the relational dynamics (e.g., *“bad relationship with her ex-husband”* [FA61]). Parenting is also a topic that often worries PM+ clients from our sample, who were often concerned about their children’s future (*“she is afraid of her daughters’ future. This is because they generally do not respect the rules set at home and at school.”* [FA11]), their relationship with them (*“A relationship with a daughter who is entering adolescence, how to keep boundaries, so as not to disturb the relationship of trust”* [FE44]*; “My youngest children are far from me”* [FA13]), or the difficulty in caring for them financially and emotionally (*“I have difficulty sending my son to school. He couldn’t go to school this year because of lack of funds.”* (FA18)*; “she cannot take care of them. So they live with their uncle.”* [FA77]).

Family problems were also commonly mentioned. With this term, we refer to problems in the relationship between members of a household living in the same house. Some of the most striking examples of such issues include “*Difference and discrimination in treatment at home between her and her older sister”* (FA15)*; “She is not accepted as a member of the family. She says she is insulted several times. Too much blame on her. She says she doesn’t know what to do to be accepted. The family does not want her to touch their things.” (*FA19). Communication and the relationship with the father of the family is also often cited *“now out of home because he quarrelled with his father.”* (MA74)*, “communication with the father due to a conflicted relationship”* (FE37). The death of a relative is an event that was not very frequently reported by clients in our sample. Among those who reported such an event in the PSYCHLOPS evaluations, family members who lost their lives were mainly husbands (*“husband died because of the current situation/war of Ethiopia.”* [FA78]), parents (*“my mother’s sudden and unexpected death”* [FA8]) as well as children (*“little boy who unfortunately died just after i gave birth.”* [FA3]).

#### 3.3.3. Theme 3: Psychosocial problems

Within this theme, we encompass all problems related purely to the dynamics within the community and economic problems. Participants reported a total of 78 times concerns related to psychosocial aspects. Specifically, 41 sentences related to community problems, 29 to financial problems, 7 to pregnancy problems and 1 to legal problems. Problems within the community concern difficult relationships with peers (*“Isolation and alienation of many friends from school and the Village, Participation in activities at school, in the village and in the community, Discrimination against school and community peers”* [FA15]), experiences of social stigma (*“stigmatisation because of his social status”* [MA24]) and participation in social and community activities (*“I find it difficult to go to my friends and participate in community activities as compared to before”* [FA26]).

Financial problems are another commonly-reported topic, either due to lack of employment (*“has no job has financial problem”* [FA69]) or generally unstable financial situations (*“extremely precarious situation with two children in his care and divorced 2 years ago the children of school age are not enrolled due to lack of funds the family can go a whole day without having anything to eat”* [MA24]), resulting in difficulties in managing basic household expenses (*“all the family’s expenses – rent, food, health, education of the children* etc. *– fall on her.”* [FA11]*; “paying a house rent is a big challenge to her.”* [FA77]).

#### 3.3.4. Theme 4: Daily activities

Participants reported a total of 40 times concerns about their daily activities. Specifically, 32 PSYCHLOPS responses related to work issues and 8 to school difficulties. With regard to work, the most discussed topics regarded relationships with colleagues (*“work relationships, which are very complex and complicated.”* [FE47]) and work status (*“he still is a trainee and with a subject to finish”* [MA1]*; “job situation not being stable enough”* [MA7]). Regarding the sphere of school instead, most of the difficulties experienced by the client’s concern performance (*“low school performance”* [FA17]*; “difficult for him to concentrate on his studies.”* [MA30]).

### 3.4. Mixed-method data

Results of the regression models indicated that there was no significant difference across identified thematic areas (general health, family relationships, psychosocial problems, daily activities) and scores across outcome measures: Thematic area on PSYCHLOPS (β = −1.80, *p* = 0.63), PHQ-9 (β = −1.11, *p* = 0.84), thematic area on GAD-7 (β = −0.27, *p* = 0.76) and thematic area on WHODAS (β = −0.17, *p* = 0.86). This indicates that the intervention was equally effective in alleviating problems described by the clients and concomitant symptoms of distress as well as functioning.

The last analysis we ran was aimed at testing whether the thematic areas varied according to the client’s region of origin. It was found that the region did not significantly predict the thematic area described by the clients (β = −0.27, *p* = 0.09).

## 4. Discussion

This paper reports a mixed-method evaluation of the impact of scale-up of the PM+ intervention beyond research-controlled settings. The evaluation included 87 participants from Sub-Saharan Africa and Eastern Europe who received the PM+ intervention as part of the MHPSS programming of the NGO SOS CVI.

The quantitative data analysis revealed that the PM+ intervention had an impact on self-reported problems and on functioning, as well as symptoms of depression and anxiety. Specifically, regarding the measures that investigate the psychological states of anxiety, depression and general disability, the average scores reflect a significant decline, shifting from the “Moderate” to “Mild” in both PHQ-9 and GAD-7. Additionally, the WHODAS score decreased from the 88th percentile to the 78th percentile. Finally, for the PSYCHLOPS we can highlight a decrease of more than 10 points.

Moreover, the clients showed significant improvements in their mental health and well-being between baseline and right after the intervention. The improvements in self-reported problems were found to be significant across all sessions, but for between pre-assessment and session 1 and session 5 and post-assessment, indicating that the changes observed between sessions are the effect of the intervention used, and not of the time of measurement or the repetition of the administration of PSYCHLOPS. The qualitative data analysis of the self-reported client problems identified four main themes of clients’ concerns: general health, family relationships, psychosocial problems and daily activities. These themes encompassed various issues such as stress, anxiety, relationship problems, financial difficulties and work-related challenges. Further analysis showed that the impact of the intervention was consistent across all identified thematic areas, suggesting that the PM+ intervention was equally effective in addressing the different types of problems described by the clients.

The study’s results align with previous findings of evaluations of PM+ in varied research-controlled settings. For instance, an RCT conducted in urban Kenya among women who had experienced gender-based violence found that PM+ was associated with moderate reductions in psychological distress and self-identified problems (Bryant et al., 2017). Specifically, the results from Bryant and colleagues indicate a moderate reduction in psychological distress at post-treatment and 3-month follow-up (Bryant et al., 2017). Furthermore, our results are in line with their findings, which indicate a reduction in self-identified problems (PSYCHLOPS) at post-treatment and 3-month follow-up in favour of PM+. Similarly, an RCT performed among Syrian refugees in the Netherlands demonstrated that PM+ effectively reduced self-identified problems and symptoms of CMHCs, including depression and anxiety (de Graaff et al., 2023). At post-assessment, PM+ had greater reductions in depression/anxiety relative to usual care, similarly to our findings. Moreover, PM+ was also found to significantly reduce self-identified problems (de Graaff et al., 2023). These trends are overall in line with our findings, in terms of the decrease in self-reported problems as well as a reduction in self-reported symptoms of mental conditions.

Similarly, the qualitative findings reported in this study align with the wider literature on the topic. Data collected within two RCTs testing the effectiveness of PM+ in Pakistan (N = 346) (Sijbrandij et al., 2015) and Kenya (N = 521) (Sijbrandij et al., 2016) was analysed to identify the most common self-reported problems faced by clients. In Pakistan, they were found to relate mostly to poor health (headaches, sleep problems, other aches and pain) and emotional problems (sadness/disappointment, anger/irritation, worries, fears). In Kenya, financial constraints (general lack of money, lack of school fees, inability to pay for basic needs, inability to develop businesses); poor health (non-specific poor health, multiple health problems, ulcers and reproductive health problems) and unemployment were most often reported by PM+ clients (Harper Shehadeh et al., 2020). This is in line with our results from the Sub-Saharan and Eastern European Regions. These themes relate to those identified in our sample, reflected in thematic areas of general health, psychosocial problems and daily activities, showing consistency across the issues experienced by PM+ clients across context and location of implementation.

The nature of the presenting problems aligns with the domains assessed by the GAD, PHQ and WHODAS instruments. For instance, stress, anxiety, relationship problems, financial difficulties and work-related challenges, which were identified as key concerns, are likely to be reflected in the measures of anxiety (GAD), depression (PHQ) and general disability (WHODAS). The assessments capture a comprehensive range of mental health and functional issues, providing a holistic view of clients’ well-being.

It is crucial to consider contextual factors that might influence the prevalence of specific challenges. Economic factors, sociopolitical conditions and cultural norms can contribute to the manifestation of certain problems. Exploring the qualitative data further or conducting additional analyses based on demographic or contextual variables may provide insights into these contextual influences.

The consistency of themes across regions (Sub-Saharan Africa and Eastern Europe) suggests that PM+ is effective in addressing common challenges regardless of cultural or regional variations. This consistency may indicate the universal applicability of the intervention in addressing fundamental human experiences and concerns.

This study has several strengths. First, it evaluates the impact of PM+ in the implementation space through an analysis of data collected in the scale-up of the intervention as part of the MHPSS programming of a large NGO. Furthermore, it offers a comprehensive assessment of its impact through a mixed-method analysis of outcomes, collected at pre- and post-assessment as well as across in session evaluations. Nevertheless, the study also holds some limitations. First, we have to highlight the lack of cultural adaptation. Furthermore, some relevant demographic information regarding, for example, the country of origin or migration history of participants was not collected. Our evaluation lacks follow-up assessments of our outcome measures, containing our ability to draw conclusions of the long-term impact of PM+ on clients. In addition, we lack data on the language in which individual helpers delivered the intervention, as well as implementation-domain factors like acceptability and feasibility, which prevent us from understanding, for example, the client-level perceptions of the intervention.

## 5. Conclusion

Taken together, these findings suggest that PM+ clients face similar daily struggles across contexts. The PM+ intervention is a promising and effective approach to address these self-reported problems and concomitant mental health symptoms and functional impairment in diverse populations and challenging settings, even beyond RCTs. The study contributes to the growing body of evidence supporting the efficacy of the PM+ methodology and underscores its potential to improve mental health outcomes for individuals facing various psychosocial difficulties. As mental health continues to be a global concern, interventions like PM+ offer valuable insights and strategies for promoting well-being and resilience in vulnerable populations.

## Data Availability

The datasets generated and analysed during the current study are available from the corresponding author on reasonable request.
