# Supplementary material for: Enhancing mental health and well-being in adults from lower-resource settings: A mixed-method evaluation of the impact of problem management plus
Source: Glob Ment Health (Camb). 2024 Apr 26;11:e56. doi: 10.1017/gmh.2024.52 (PMC11094550; doi:10.1017/gmh.2024.52)
Supplement: Peer review history

# Editor report: Recommendation: Enhancing mental health and well-being in adults from lower-resource settings: A mixed-method evaluation of the impact of problem management plus — R0/PR2

**DOI:** 10.1017/gmh.2024.52.pr2

**Affiliations:** Public Health, Section of Global Health, University of Copenhagen, Denmark

No accompanying comment.

# Editor report: Decision: Enhancing mental health and well-being in adults from lower-resource settings: A mixed-method evaluation of the impact of problem management plus — R0/PR3

**DOI:** 10.1017/gmh.2024.52.pr3

**Affiliations:** London School of Hygiene & Tropical Medicine, United Kingdom of Great Britain and Northern Ireland

No accompanying comment.

# Editor report: Recommendation: Enhancing mental health and well-being in adults from lower-resource settings: A mixed-method evaluation of the impact of problem management plus — R1/PR5

**DOI:** 10.1017/gmh.2024.52.pr5

**Affiliations:** Public Health, Section of Global Health, University of Copenhagen, Denmark

No accompanying comment.

# Editor report: Decision: Enhancing mental health and well-being in adults from lower-resource settings: A mixed-method evaluation of the impact of problem management plus — R1/PR6

**DOI:** 10.1017/gmh.2024.52.pr6

**Affiliations:** London School of Hygiene & Tropical Medicine, United Kingdom of Great Britain and Northern Ireland

No accompanying comment.

# Editor report: Recommendation: Enhancing mental health and well-being in adults from lower-resource settings: A mixed-method evaluation of the impact of problem management plus — R2/PR8

**DOI:** 10.1017/gmh.2024.52.pr8

**Affiliations:** Public Health, Section of Global Health, University of Copenhagen, Denmark

No accompanying comment.

# Editor report: Decision: Enhancing mental health and well-being in adults from lower-resource settings: A mixed-method evaluation of the impact of problem management plus — R2/PR9

**DOI:** 10.1017/gmh.2024.52.pr9

**Affiliations:** London School of Hygiene & Tropical Medicine, United Kingdom of Great Britain and Northern Ireland

No accompanying comment.
